# Supplementary material for: Loss of function of metabolic traits in typhoidal Salmonella without apparent genome degradation
Source: mBio. 2024 Apr 4;15(5):e00607-24. doi: 10.1128/mbio.00607-24 (PMC11077982; doi:10.1128/mbio.00607-24)
Supplement: Supplemental Figures — Figures S1 to S16. [file mbio.00607-24-s0001.pdf]

Supplementary Information

Loss of function of metabolic traits in typhoidal *Salmonella* without apparent genome degradation

Leopoldo F. M. Machado and Jorge E. Galán*
Department of Microbial Pathogenesis, Yale School of Medicine, New Haven, CT0653

Key words: Salmonella pathogenesis, bacterial metabolism, bacterial evolution, pseudogenes, biosensors, *Salmonella* Typhi, *Salmonella* Paratyphi A, convergent evolution

*: for correspondence: jorge.galan@yale.edu

Supplementary figures

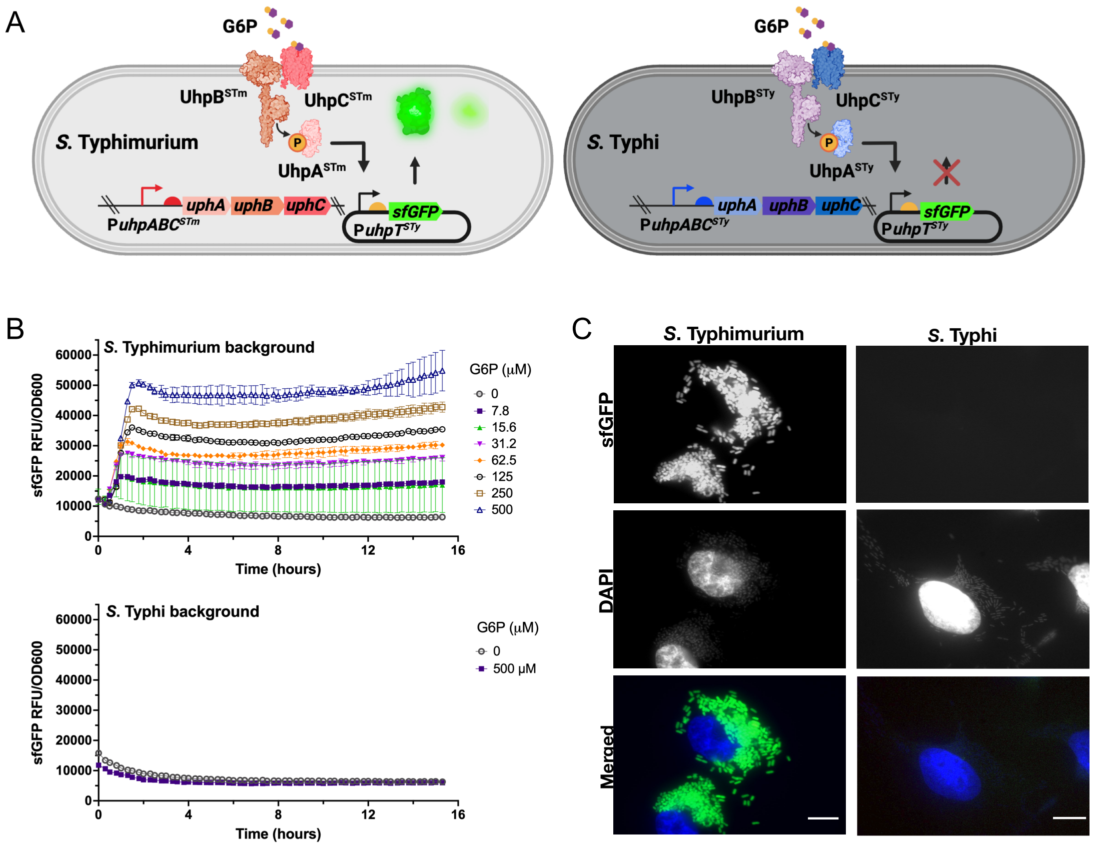

Figure S1. (**A**) Diagram of the G6P Biosensor for detection of glucose-6-phosphate in *S.* Typhimurium and *S.* Typhi. The membrane UhpC sensor detects G6P on the extracellular environment and interacts with the membrane UhpB histidine kinase that phosphorylates the cytosolic UhpA response regulator, which activates the P*uhpT* promoter. The *S.* Typhi P*uhpT* promoter was coupled to a *sfGFP* reporter gene on a plasmid system and transformed into *S.* Typhimurium and *S*. Typhi. In the presence of G6P, the cognate promoter P*uhpT*^{STy} is activated leading to sfGFP expression. The possible steps where the sensing and regulation could be impaired in *S*. Typhi are noted. (**B**) Test of the response of the *S*. Typhimurium and *S*. Typhi G6P biosensors after growth in media containing increasing concentrations of G6P. The sfGFP fluorescense signal over time for the *S*. Typhimurium (upper graph) and *S*. Typhi (lower graph) G6P biosensors are shown. Values indicate the mean ± SEM of n= 3 replicates per condition. (**C**) Functionality of the G6P Biosensor in *S.* Typhimurium and *S.* Typhi in the context of cultured epithelial cell infection. Fluorescence microscopy of HeLa cells infected with bacterial strains encoding the G6P biosensors. HeLa cells were infected with wild type *S*. Typhimurium and *S*. Typhi strains encoding the G6P biosensors for 20 hs, fixed, stained with DAPI, and examined under a fluorescence microscope. For all images, brightness and contrast were adjusted for each of the individual channels to maximize visual clarity using the same parameters for both strains. Scale bars, 10 µm.

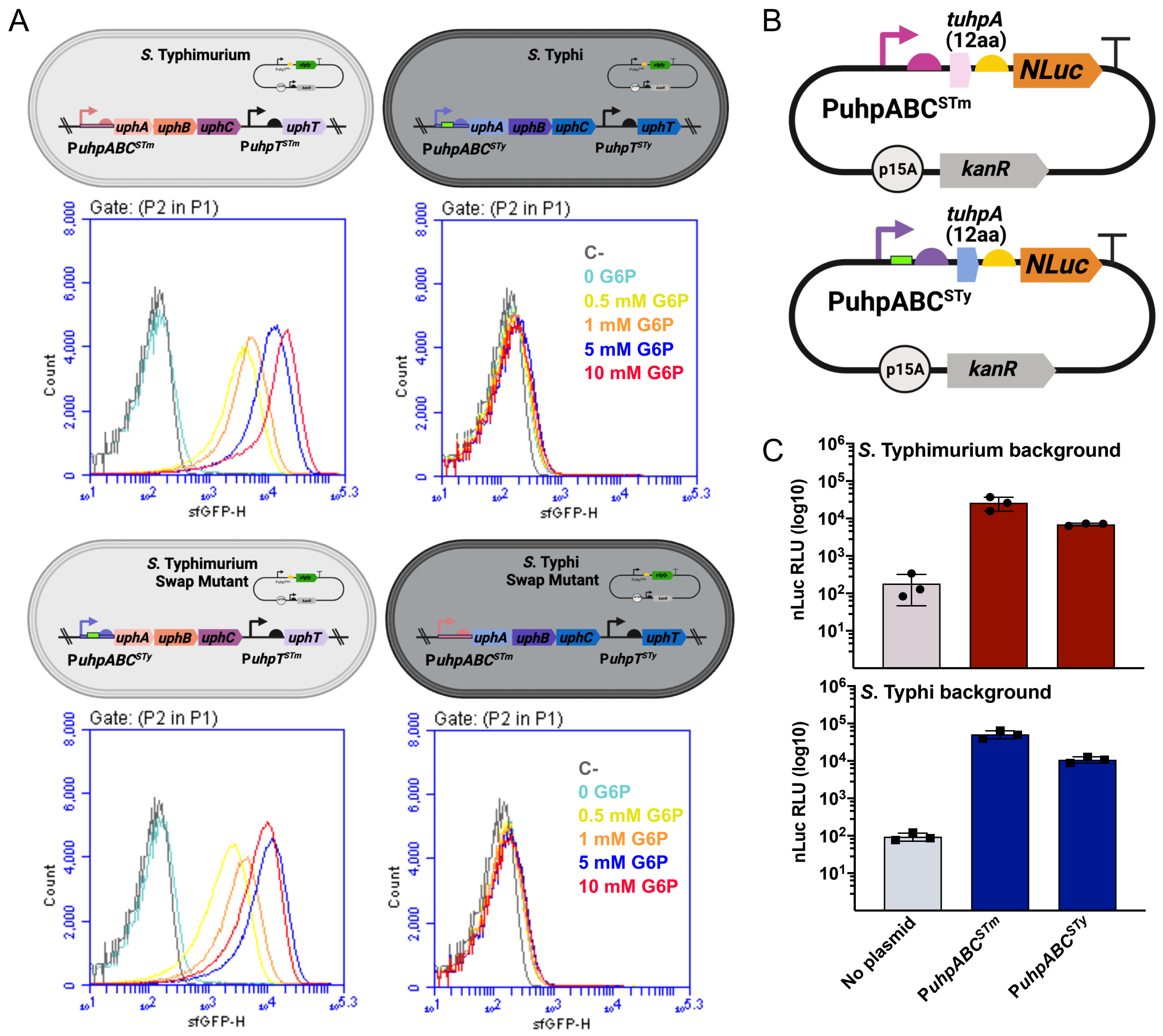

Figure S2. Investigation of the *S*. Typhi P*uhpABC* promoter. (**A**) Wild-type and mutant strains of *S*. Typhimurium containing the P*uhpABC* promoter from *S*. Typhi (P*uhpABC*^{STy}), or *S*. Typhi containing the P*uhpABC* promoter from *S*. Typhimurium (P*uhpABC*^{STm}), both harboring the G6P biosensor (P*uhpT*-sfGFP) were grown in presence of G6P for 20 hs and analyzed by flow-cytometry. Histograms show the sfGFP fluorescence intensities of individual bacteria for the indicated concentration of G6P. (**B**) Diagram of the transcriptional reporters in which the P*uhpABC*^{STm} or the P*uhpABC*^{STy} (containing an 80 bp insert indicated with a green rectangle) promoters drive the expression of a NanoLuc luciferase (NLuc). (**C**) *S*. Typhimurium and *S*. Typhi harboring the P*uhpABC*^{STm}-*NLuc* or P*uhpABC*^{STy}-*NLuc* reporters were grown for 20 hs, lysed and the luminescence was measured on a microplate reader. The signal in Relative Luminescence (RLU) for the indicated strains is shown. No statistically significative difference was observed between the P*uhpABC*^{STm}-*NLuc* or P*uhpABC*^{STy}-*NLuc* reporters as determined by Anova with Dunnett's multiple comparisons test.

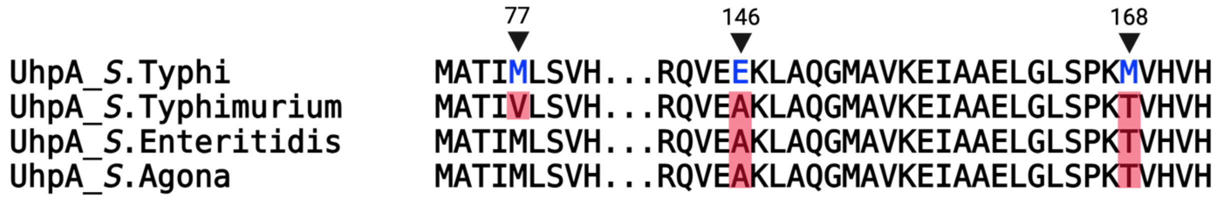

Figure S3. Alignment of portions of the amino acid sequence of the transcriptional regulator UhpA showing differences between *S*. Typhi CT18 and nontyphoidal *S. enterica* serovars *S.* Typhimurium LT2, *S.* Enteritidis P125109 and *S.* Agona SL483. The rest of the sequences not shown are identical between the different serovars.

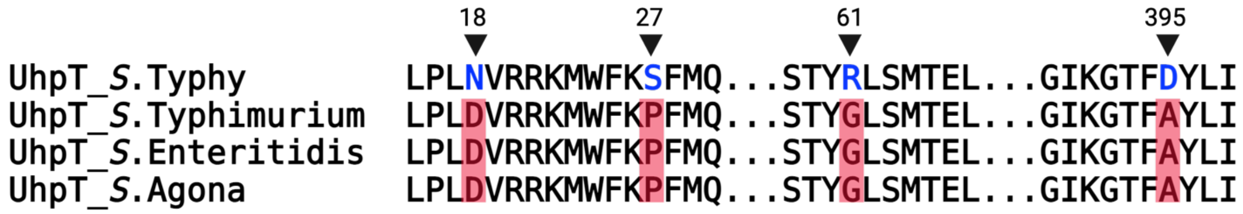

Figure S4. Alignment of portions of the amino acid sequence of the antiporter UhpT showing differences between *S*. Typhi CT18 and nontyphoidal *S. enterica* serovars *S.* Typhimurium LT2, *S.* Enteritidis P125109 and *S.* Agona SL483. The rest of the sequences not shown are identical between the different serovars.

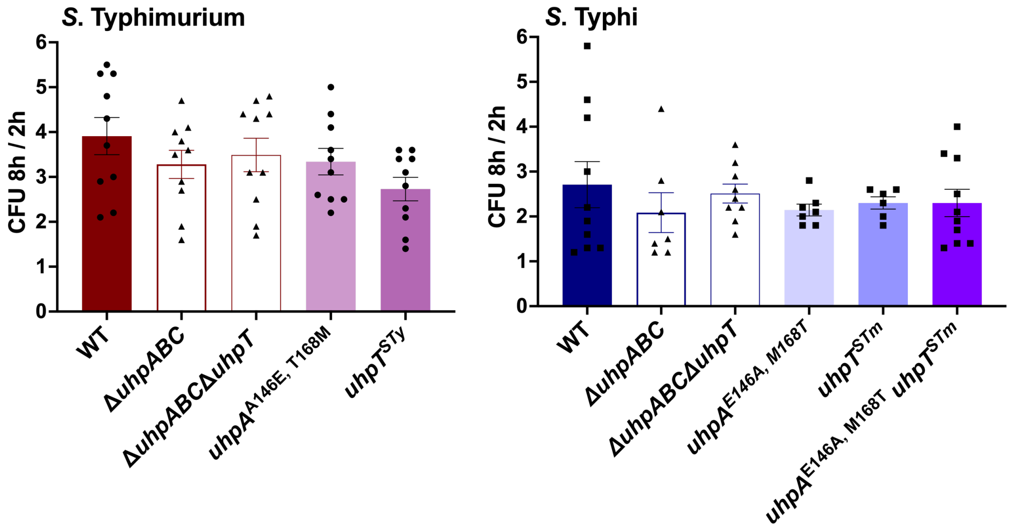

Figure S5. Intracellular growth of different *S*. Typhimurium or *S.* Typhi *uhp* mutants. Cultured HeLa cells were infected with indicated bacterial strains and the CFU 2 and 8 hours after infection were determined by plate dilutions. Values represent the ratio between the CFUs measured at 8 and 2 hours post infection and are the mean ± SEM of n= 7 - 10 replicates per condition. No statistically significative difference was observed as determined using Anova with Dunnett's multiple comparisons test.

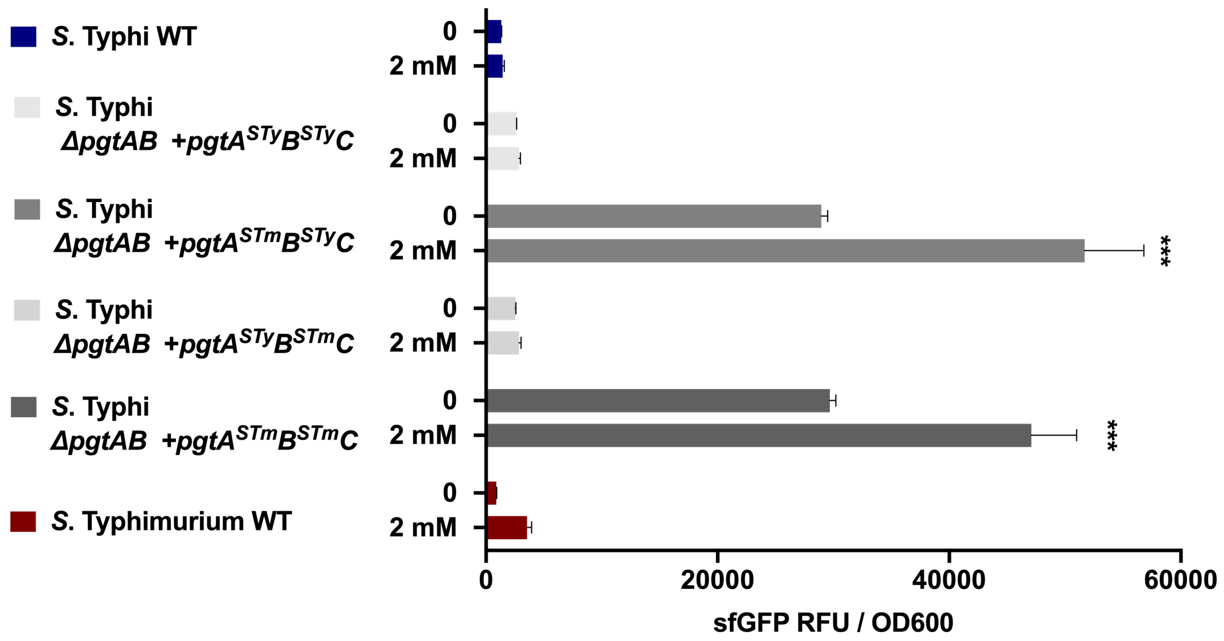

Figure S6. Investigation of the functionality of the PgtA and PgtB regulatory proteins. The *S.* Typhi *pgtA* and *pgtB* genes were swapped for their *S*. Typhimurium homologs in the 3PG biosensor plasmid. The resulting constructs were introduced into the *S*. Typhi *ΔpgtAB* mutant background and tested on the presence or absence of 3PG at 2 mM. The fluorescent gene expression normalized to cell density (sfGFP RFU / OD600) is shown. Values indicate the mean ± SEM of n= 3 replicates per condition. Asterisks denote statistically significant differences relative to the corresponding uninduced sample determined using Anova with Dunnett's multiple comparisons test. ***p < 0.001.

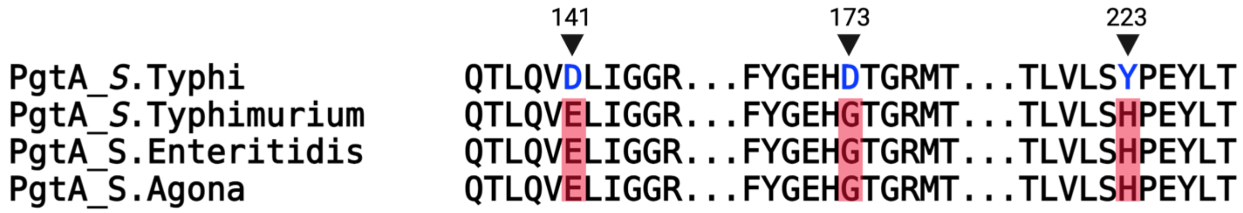

Figure S7. Alignment of portions of the amino acid sequence of the transcriptional regulator PgtA showing differences between *S*. Typhi CT18 and nontyphoidal *S. enterica* serovars *S.* Typhimurium LT2, *S.* Enteritidis P125109 and *S.* Agona SL483. The rest of the sequences not shown are identical between the different serovars.

A

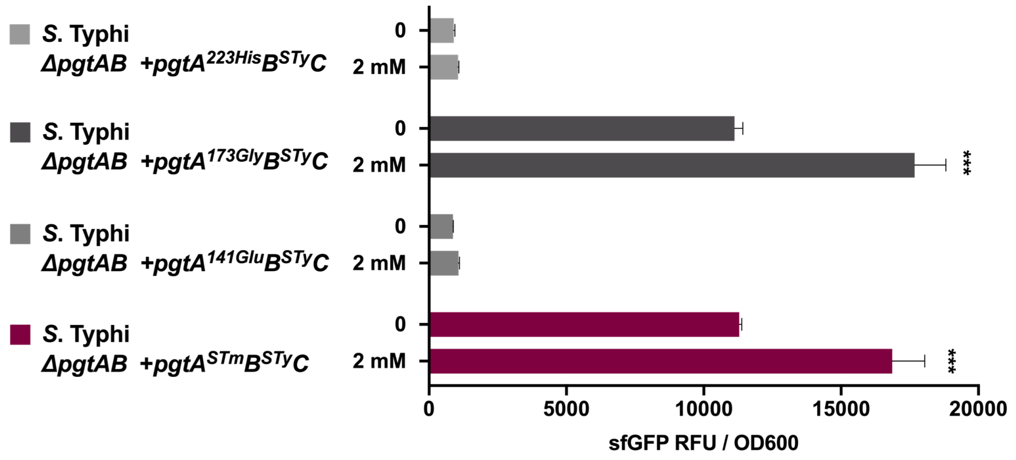

B

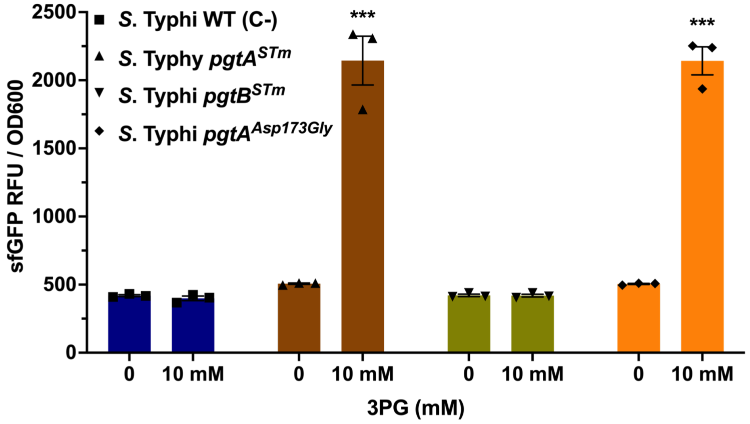

Figure S8. Investigation of the functionality of *S*. Typhi PgtA amino acid substitutions**.** (**A**) *S*. Typhi PgtA amino acids at the indicated positions were substituted for the equivalent *S*. Typhimurium residues in the plasmid backbone containing the 3PG biosensor. The resulting constructs were introduced into the *S*. Typhi *ΔpgtAB* mutant background and the transcriptional response was tested in the presence or absence of 3PG at 2mM. Values represent the fluorescence signal normalized to cell density (sfGFP RFU / OD600) and are the mean ± SEM of n= 4 replicates per condition. (**B**) The S. Typhi *pgtA* or *pgtB* were individually swapped for their S. Typhimurium homologues (S. Typhi *pgtA*^{Stm} and S. Typhi *pgtB*^{Stm}*)*. The resulting strains and an *S*. Typhi strain encoding the *pgtA*^{Asp173Gly}*,* all encoding the 3PG biosensor, were tested for their transcriptional response to 3PG at 10 mM. The fluorescence signal normalized to cell density (sfGFP RFU / OD600) is shown. Values indicate the mean ± SEM of n= 3 replicates per condition. For all panels, asterisks denote statistically significant differences relative to the corresponding uninduced sample determined using Anova with Dunnett's multiple comparisons test. ***p < 0.001.

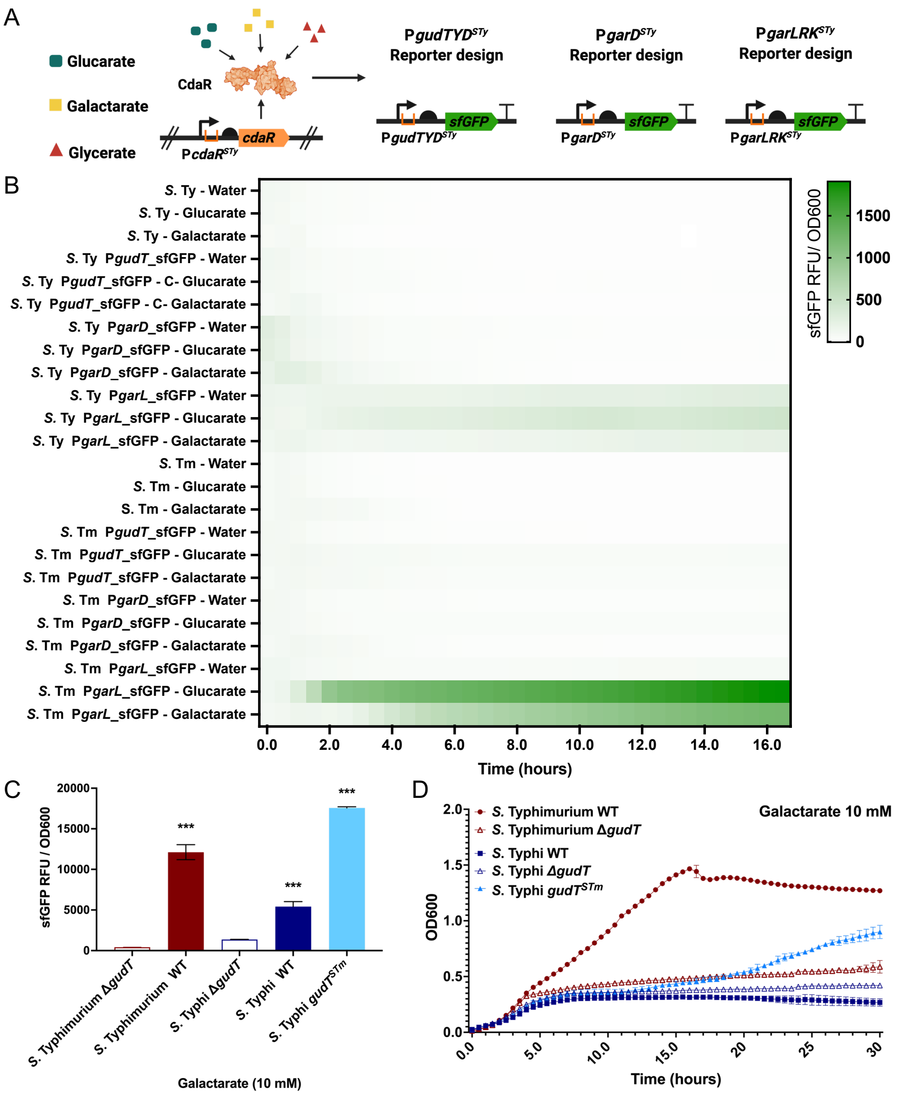

Figure S9. (**A**) (**A**) Diagram of the biosensor for the detection of glucarate, galactarate and glycerate based on the CdaR transcription factor and the cognate promoters P*gudTYD*, P*garD* and P*garLRK* driving expression of *sfGFP*. (**B**) The indicated wild-type strains of *S*. Typhimurium (S. Tm) and *S*. Typhi (S. Ty) harboring the indicated biosensors were tested in LB medium containing glucarate or galactarate (1 mM), as indicated, or water as a negative control, and the fluorescence signal (normalized to cell density) was measured (sfGFP RFU/OD600) for each strain. (**C**) The indicated *S*. Typhimurium and *S*. Typhi mutant strains all harboring the P*garL_sfGFP* transcriptional reporter were grown in LB medium containing galactarate at 10mM, and the fluorescence signal (normalized to cell density) was measured (sfGFP RFU/OD600) for each strain. Values are the mean ± SEM of n= 3 replicates per condition. Asterisks denote statistically significant differences determined using Anova with Dunnett's multiple comparisons test. ***p < 0.001. (**D**) Growth kinetics of wild-type *S.* Typhimurium and *S.* Typhi or their isogenic mutants *ΔgudT, ΔgudT* and *gudT*^{STm} as indicated. Growth was monitored in M9 +0.05% casamino acids medium containing 10 mM galactarate as the only carbon source. The OD600 was measured at 30 min intervals and values represent the mean ± SEM of n=3-4 replicates per condition.

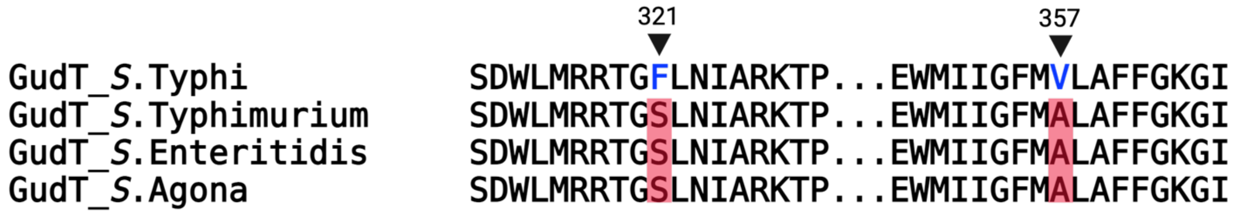

Figure S10. Alignment of portions of the amino acid sequence of the glucarate/galactarate GudT transporter showing differences between *S*. Typhi CT18 and nontyphoidal *S. enterica* serovars *S.* Typhimurium LT2, *S.* Enteritidis P125109 and *S.* Agona SL483. The rest of the sequences not shown are identical between the different serovars.

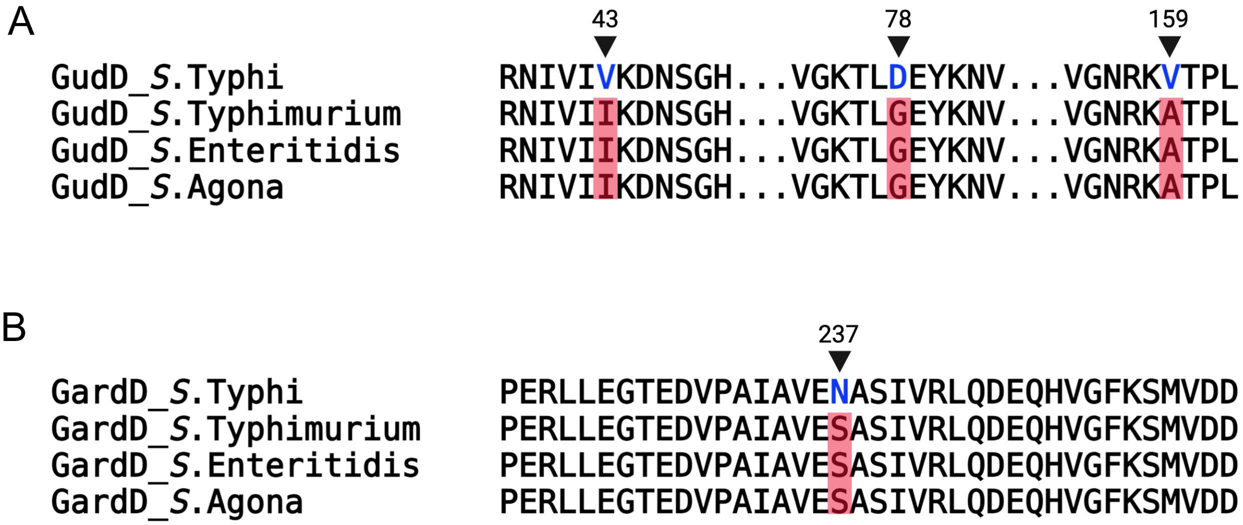

Figure S11. Alignment of portions of the amino acid sequences of the GudD (A) and GarD (B) showing differences between *S.* Typhi CT18 and nontyphoidal *S. enterica* serovars *S.* Typhimurium LT2, *S.* Enteritidis P125109 and *S.* Agona SL483. The rest of the sequences not shown are identical between the different serovars.

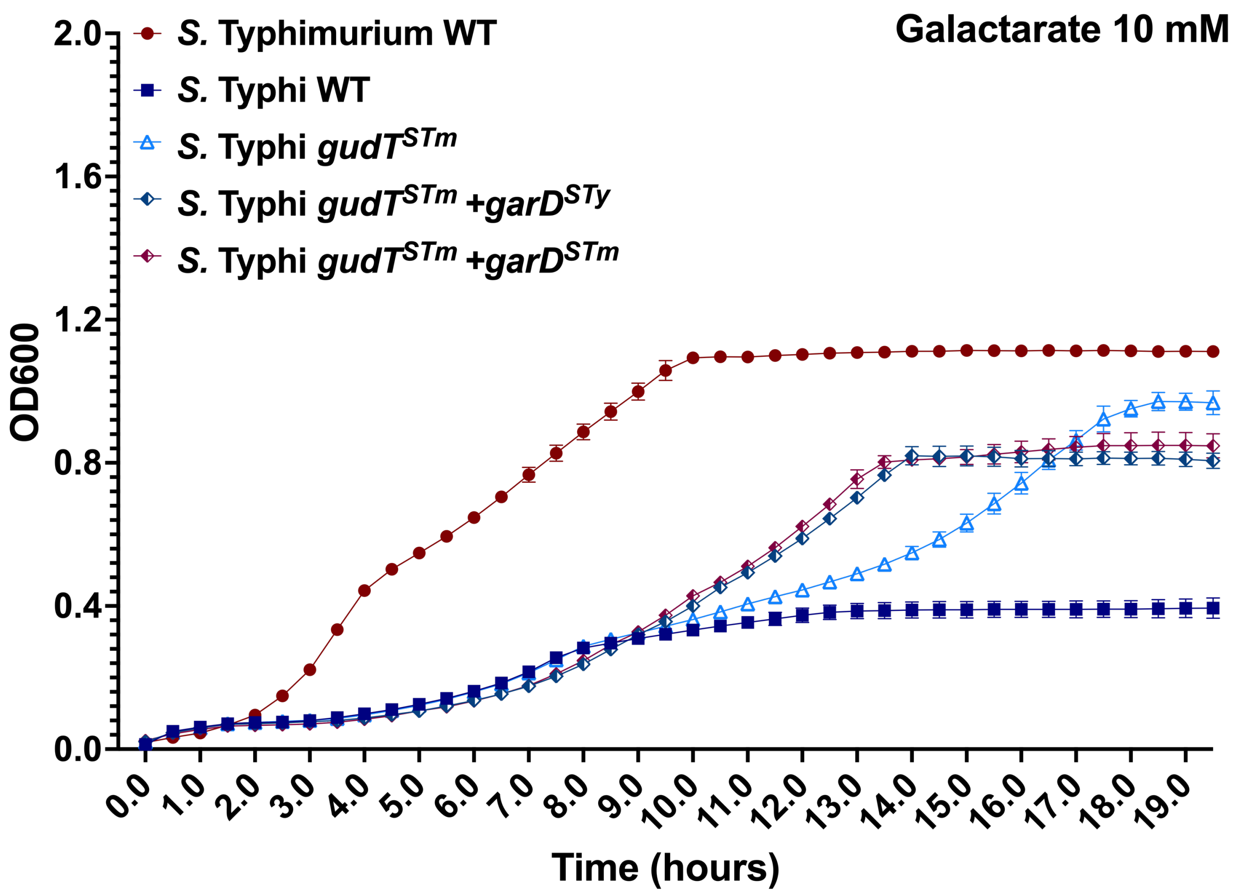

Figure S12. Growth kinetics of *S.* Typhi strains expressing *S.* Typhimurium *gudT* (*gudT*^{STm}) and *garD* (*garD*^{STm}) or (*garD*^{STy}) alleles as indicated. Strains were grown in M9 +0.05% casamino acids medium containing 10 mM galactarate as the only carbon source, and the OD600 was measured at 30 min intervals. Values indicate the mean ± SEM of n= 3-4 replicates per condition.

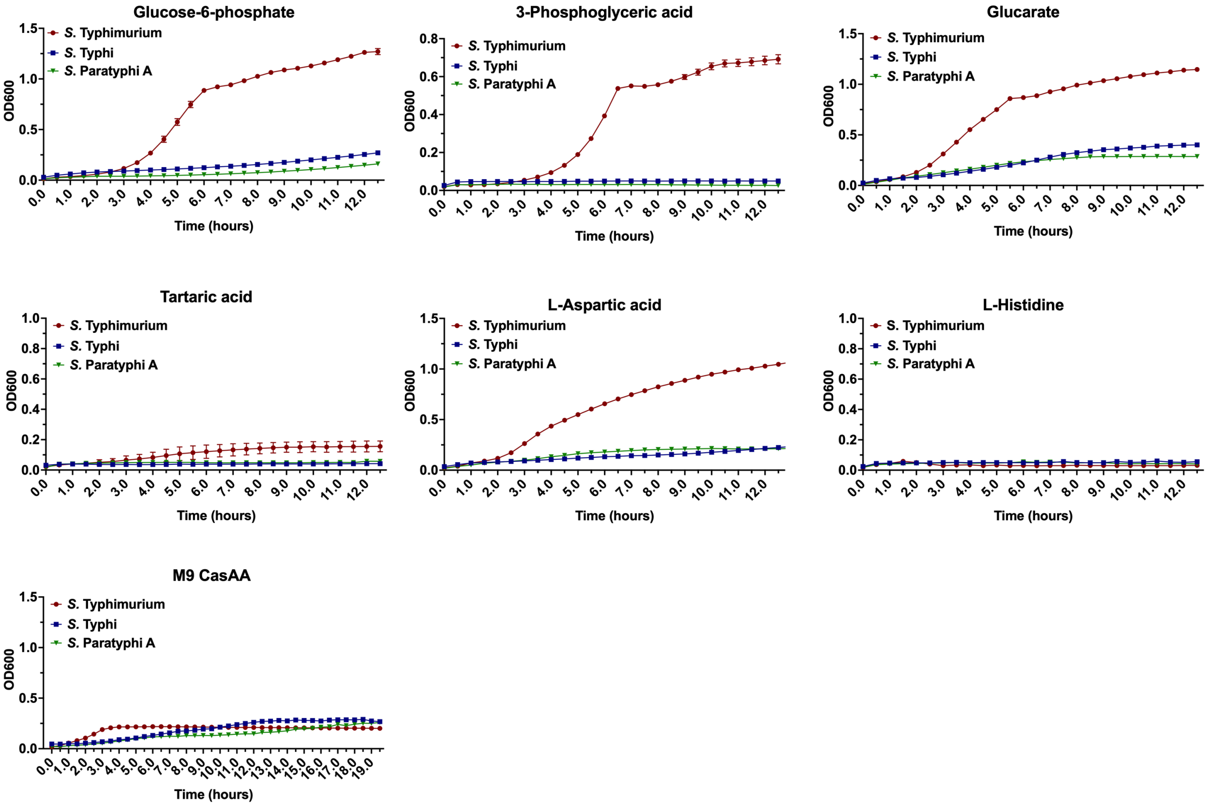

Fig S13. Growth kinetics of *S*. Typhimurium, *S*. Typhi and *S*. Paratyphi A in minimal medium containing glucose-6-phosphate, 3-phosphoglyceric acid, glucarate, tartrate, aspartate, or L-histidine as the sole carbon source. Bacteria were grown in M9 medium supplemented with casamino acids (0.05%) and containing 20 mM of the different metabolites and the OD600 was measured at 30 min intervals. Values indicate the mean ± SEM of n= 3-4 replicates per condition.

A

```
                                      212
                                       ▼
STY3536_S.Typhi          GEVGSVGEQRDYSHVPQWKQRLSLVVLIATI
STM3356_S.Typhimurium    GEVGSVGVQRDYSHVPQWKQRLSLVVLIATI
SEN3189_S.Enteritidis    GEVGSVGEQRDYSHVPQWKQRLSLVVLIATI
SAG3547_S.Agona          GAVGSIGEQHDYSHVPRWKQLLSLIVLIVTI
```

B

```
                                               97     104
                                                ▼      ▼
STY3537_S.Typhi          LESEAVAKASRPGTDISRIAEVHYAAEKALA
STM3357_S.Typhimurium    LESEAVAKASRPGSDISRIAQVHYAAEKALA
SEN3190_S.Enteritidis    LESEAVAKASRPGTDISRIAQVHYAAEKALA
SAG3548_S.Agona          LESEAVAKASRPGTDISRIAQVHYAAEKALA
```

C

```
                                                 35
                                                  ▼
STY3538_S.Typhi          EILSGNIKAGEELAQGSIAEQLGLSRMPVRE
STM3358_S.Typhimurium    EILSGNIKAGEELAQESIAEQLGLSRMPVRE
SEN3191_S.Enteritidis    EILSGNIKAGEELAQESIAEQLGLSRMPVRE
SAG3549_S.Agona          EILSGNIKAGEELAQECIAEQLGLSRMPVRE
```

Figure S14. Alignment of portions of the amino acid sequence of *S*. Typhi STY3536 (**A**), STY3537 (**B**), and STY3538 (**C**) showing differences between *S*. Typhi CT18 and nontyphoidal *S. enterica* serovars *S.* Typhimurium LT2, *S.* Enteritidis P125109 and *S.* Agona SL483. The rest of the sequences not shown are identical between the different serovars.

A

```
                          69             84 86       240       274
                           ▼              ▼ ▼         ▼         ▼
DcuS_S.Typhi          SPAIREGLKKPPAESGIQPLSEAV...AVDGSGE...HAWPQVV
DcuS_S.Typhimurium    SPAVREGLKKPPAESGIQTLAEAV...AVDDSGE...HAWSQVV
DcuS_S.Enteritidis    SPAIREGLKKPPAESGIQPLAEAV...AVDDSGE...HAWSQVV
DcuS_S.Agona          SPAVREGLKKPPAESGIQTLAEAV...AVDDSGE...HAWSQVV
```

B

```
                                                   269
                                                    ▼
tar_S.Typhi           NEIGELAGTVEHMQRSLIDTVTQVCEGSDAIYSGTSEIAAGNTD
tar_S.Typhimurium     NEIGELAGTVEHMQRSLIDTVTQVREGSDAIYSGTSEIAAGNTD
tar_S.Enteritidis     NEIGELAGTVEHMQRSLIDTVTQVREGSDAIYSGTSEIAAGNTD
tar_S.Agona           NEIGELAGTVEHMQRSLIDTVTQVREGSDAIYSGTSEIAAGNTD
```

C

```
                            67          198-9                387
                             ▼           ▼▼                   ▼
AsnB_S.Typhi          PLYNAKKTHVL...QRDWFDYDAVKDNVTD...HMYDCTRANKA
AsnB_S.Typhimurium    PLYNARKTHVL...QRDWFEFDAVKDNVTD...HMYDCARANKA
AsnB_S.Enteritidis    PLYNARKTHVL...QRDWFDYDAVKDNVTD...HMYDCARANKA
AsnB_S.Agona          PLYNARKTHVL...QRDWFDYDAVKDNVTD...HMYDCARANKA
```

Figure S15. Alignment of portions of the amino acid sequence of *S*. Typhi DcuS (**A**), Tar (**B**), and AsnB (**C**) showing differences between *S*. Typhi CT18 and nontyphoidal *S. enterica* serovars *S.* Typhimurium LT2, *S.* Enteritidis P125109 and *S.* Agona SL483. The rest of the sequences not shown are identical between the different serovars.

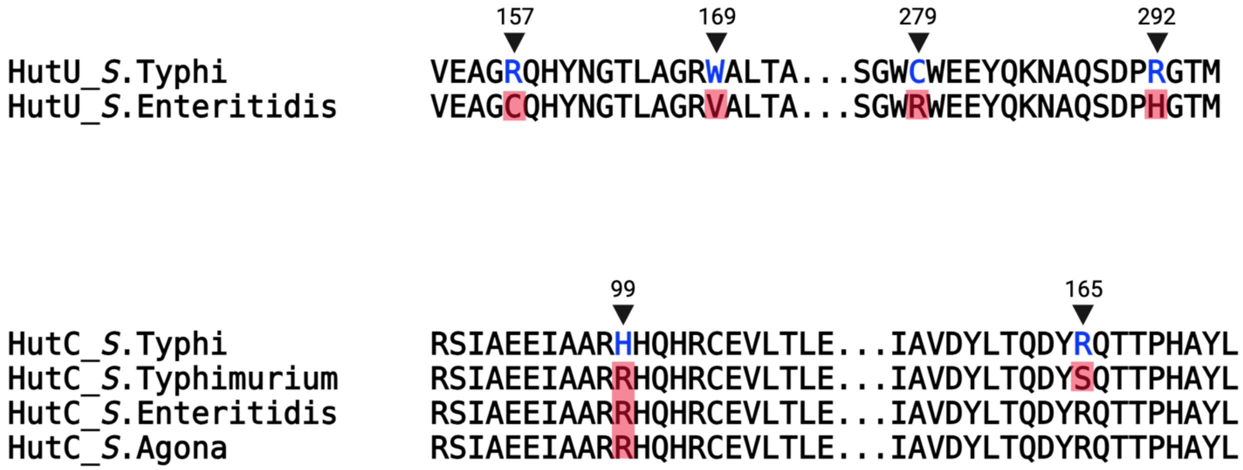

Figure S16. Alignment of portions of the amino acid sequence of *S*. Typhi HutU (**A**) and HutC (**B**) showing differences between *S*. Typhi CT18 and nontyphoidal *S. enterica* serovars *S.* Typhimurium LT2, *S.* Enteritidis P125109 and *S.* Agona SL483. The rest of the sequences not shown are identical between the different serovars.
